# Clinicopathological evaluation of 164 dental follicles and dentigerous cysts with emphasis on the presence of odontogenic epithelium in the connective tissue. The hypothesis of “focal ameloblastoma”

**DOI:** 10.4317/medoral.18372

**Published:** 2012-10-20

**Authors:** Marco Meleti, Isaäc van der Waal

**Affiliations:** 1 DDS, PhD, Consultant Professor Unit of Oral Pathology and Laser-assisted Surgery, Department of Otolaryngological/Dental/Ophthalmological and Cervico-Facial Sciences, University of Parma, Italy. Department of Oral and Maxillofacial Surgery/Oral Pathology, VU medical center/ACTA, Amsterdam, The Netherlands; 2 DDS, PhD, Full Professor, Head Department of Oral and Maxillofacial Surgery/Oral Pathology, VU medical center/ACTA, Amsterdam, The Netherlands; 3 ….

## Abstract

Objectives: Some ameloblastomas presumably originate from odontogenic epithelium within the connective tissue of dental follicles and dentigerous cysts. Therefore, it would seem reasonable to discuss as whether odontogenic epithelium proliferations, frankly displaying ameloblastomatous features (“focal ameloblastoma”), should be considered as an “early” ameloblastoma. Study Design: Histopathological reports from 164 dental follicles and dentigerous cysts from the Department of Oral and Maxillofacial Surgery/Oral Pathology of the VU Free University medical center in Amsterdam, The Ne-therlands, were reviewed. Histopathological slides from 39 cases reporting the presence of odontogenic epithelium within the connective tissue were re-evaluated in order to assess the possible presence of focal ameloblastomas. Results: Focal ameloblastomas were detected in one dental follicle and in two dentigerous cysts. During a follow-up period of 6, 8 and 22 years, respectively, no clinical signs of (recurrent) ameloblastoma have occurred in these patients. Conclusions: Focal ameloblastoma possibly represents the early stage of ameloblastoma development.

** Key words:**Ameloblastoma, odontogenic epithelium, dentigerous cyst, dental follicle.

## Introduction

The dental follicle (DF) consists of tissues derived from neural crest cells and forms the ectomesenchymal portion of a tooth germs (1). Histologically, DFs are characterized by fibrous connective tissue with variable amounts of lining epithelium, including enamel (columnar), cuboidal, squamous and, rarely, respiratory epithelium (1-5). The type of lining epithelium seems to be related to the patient’s age (5). According to Stanley et al. the enamel epithelium is most frequently observed in specimens from people younger than 21 years while the stratified squamous epithelium is almost exclusively found in patients older than 26 (5). Myxoid changes and calcifications may also be present (1,3).

A dentigerous cyst (DC) encloses the crown of an unerupted tooth by expansion of its DF and it is attached to the dentoenamel junction (6). The DC is the second most common odontogenic cyst within the oral cavity, the first being the radicular cyst (7). Histopathologically, DC is characterized by squamous stratified epithelium without formation of rete ridges. Mild to severe diffuse chronic inflammatory infiltrate, Russell bodies and cholesterol clefts are frequently observed (8).

According to Saravana, the presence of squamous epithelium in the lining of a tissue sac that invests the crown of an unerupted or impacted tooth defines progression from DF to DC (9).

The presence of odontogenic epithelium (OE) remnants within the connective tissue of DFs as well as in the wall of DCs is a well-known phenomenon (1,10). Kim and Ellis reported islands of OE embedded in the connective tissue of DFs in 79% of the 847 cases they reviewed (1). It is generally thought that these OE remnants are inactive and do not have clinical significance (9). Nevertheless, some ameloblastomas presumably originate from OE islands within the wall of a DC (so-called “mural ameloblastoma”) (10-12).

How much OE proliferation is actually needed for classifying a lesion as ameloblastoma is unknown (10). It is, indeed, questionable whether a few islands of OE proliferation, frankly displaying ameloblastoma features (here called “focal ameloblastoma”; FA), should be considered as an “early” ameloblastoma or, instead, that they represent a separated nosological entity lacking neoplastic activity.

Here we report a clinico-pathological evaluation of 164 DFs and DCs of the files of the Department of Oral and Maxillofacial Surgery/Oral Pathology of the VU Free University medical center in Amsterdam, The Netherlands, with emphasis on the detec-tion of OE and possibly FAs in the connective tissue.

## Material and Methods

Histopathological reports of cases classified as DFs and/or DCs in the database of the Department of Oral and Maxillofacial Surgery/Oral Pathology of the VU Free University medical center in Amsterdam, The Netherlands, were retrieved and reviewed. The search disclosed 164 lesions obtained from 154 patients (56 males, 98 females; male to female ratio = 1:1.75; mean age 24.3 years) recorded between 1985 and 2004. Information on the age, gender, teeth involved and clinical diagnosis were obtained. Histopathological slides from cases reporting the presence of OE within the connective tissue were retrieved and reevaluated in order to assess the possible presence of FAs. All the tissues had been formalin fixed and stained with hematoxylineosin.

In case of FA detection, patient pathological history was reviewed through the nationwide network and Registry of histo and cytopathology (PALGA) in the Netherlands in order to evaluate if a (recurrent) ameloblastoma was recorded.

## Results

-Age, gender and location

One-hundred and thirty out of 164 (79.3%) specimens obtained from 123 patients (47 males, 76 females; male to female ratio = 1:1.6; mean age 23.2 years) were classified as DFs (DF group). Lesions diagnosed as DCs (DC group) were 23 (14%) and they were removed from 22 patients (6 males and 16 females, mean age 35.7 years). For 11 out 164 cases (6.7%), the diagnosis was questionable since focal areas of stratified squamous epithelium suggestive of DC, were observed within the context of a DF ( Table 1).

**Table 1:**
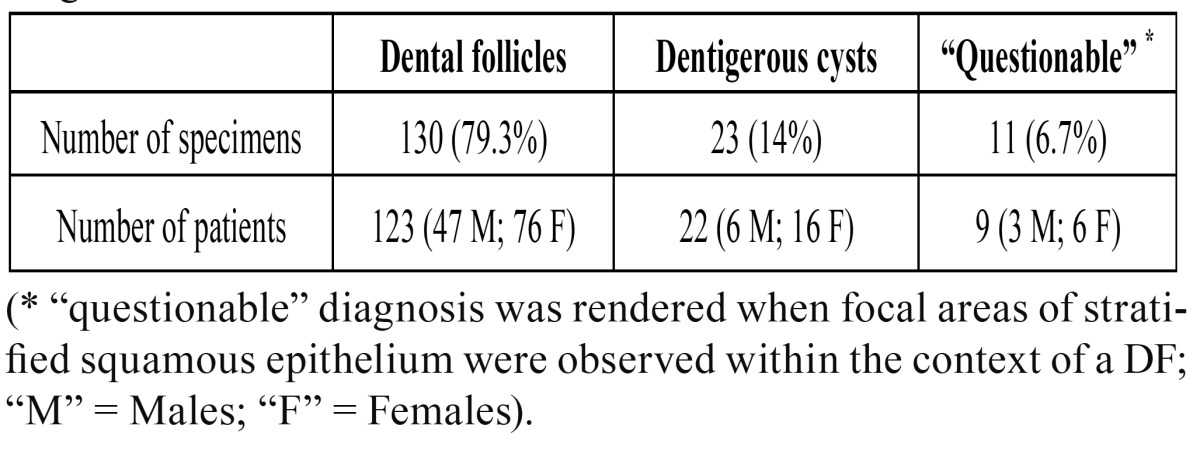
Subclassification of 164 cases according to histopatological diagnosis.

Among 154 patients, 76 (43.3%) and 51 (31.1%) were in the second and in the third decade of life, respectively.

Seventy-eight percent (128 out of 164) of the specimens were associated with lower impacted third molar teeth (66 cases associated to 4.8; 62 cases associated to 3.8) while 4.3% (7 out of 164) and 3.6% (6 out of 164) were associated with upper canine and upper third molar teeth, respectively.

Ninety-eight out of 130 cases (75.3%) in the DF group and 21 out of 23 lesions (91.3%) of the DC group were associated with lower impacted third molars (DF group: 50 cases associated to 3.8; 48 cases associated to 4.8. DC group: 13 cases associated to 4.8; 8 cases associated to 3.8). For 5 cases in the DF group no data on location were available.

-Clinical versus histopathological diagnosis

One-hundred and two out 130 specimens (78.5%) classified as DFs were submitted for histopathological evaluation with a clinical diagnosis of “dental follicle” or “enlarged follicle” associated with an impacted tooth. In one of these cases, a second working diagnosis of “ameloblastoma” was mentioned, while in 2 cases the possibility of a DC was taken into account in the differential diagnosis. In 20 out of 130 cases (15.4%) the clinical diagnosis was “dentigerous cyst” or “follicular cyst” and therefore, not in accordance with the histopathological diagnosis. In one case the clinical diagnosis was keratocyst. Seven surgical specimens were submitted without a working diagnosis.

Fifteen out of 23 lesions (65.2%) histopathologically classified as DCs were submitted with a clinical diagnosis of “dental follicle” while only 6 lesions (26.1%) of the same group had a clinical diagnosis of “dentigerous cyst”. One surgical specimen was provided with a working diagnosis of ameloblastoma while another was reported as “fibrous tissue around the crown of an im-pacted tooth”. Among the group of the histopathologically “questionable” lesions, 8 and 2 out of 11 had a clinical diagnosis of “dental follicle” and “dentigerous cyst”, respectively. One specimen was provided without a working diagnosis.

-Histopathological findings

**Figure 1:**
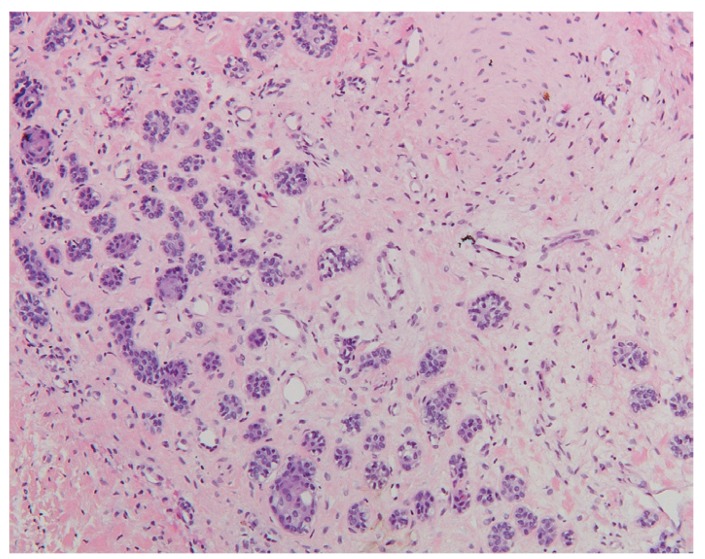
Proliferation of normal odontogenic epithelium in a dental follicle (H.E.; orig. magn. x100).

**Figure 2:**
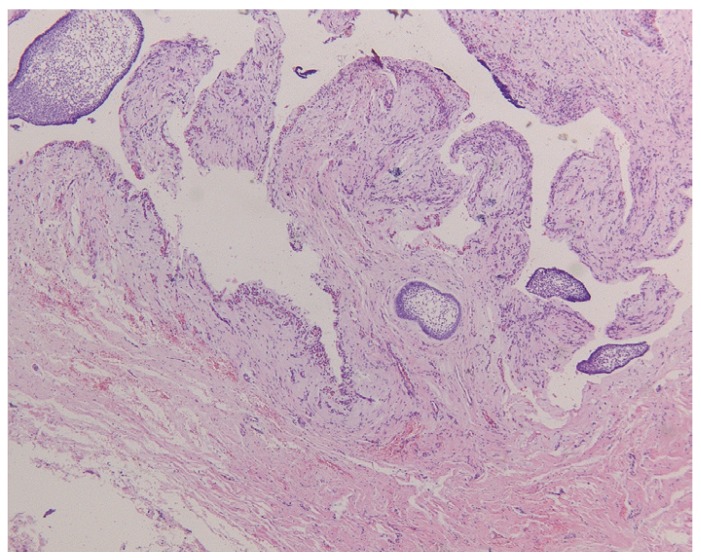
Foci of ameloblastomatous epithelium in a dental follicle (H.E.; orig. magn. x25).

**Figure 3:**
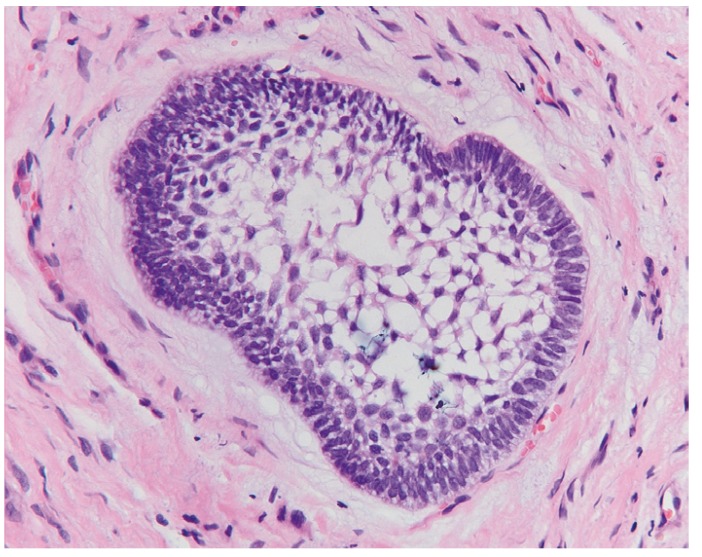
High power view of ameloblastomatous island (H.E.; orig. magn. x200).

The most frequent histopathological description of DF includes presence of connective fibrous tissue partially or completely lined by columnar or cuboidal epithelium, representing the reduced epithelium from the enamel organ. Four cases presented distinct myxoid changes of the connective tissue while in 47 cases (36.1%) presence of a chronic inflammatory infiltrate, ranging from mild to severe, was reported. Reactive and hyperplastic changes of the epithelium without cellular atypia were described in 6 cases. Two cases were characterized by the presence of focal calcifications. Small OE remnants in the connective tissue were reported in 27 DFs (Fig. 1). Histopathological reevaluation of the specimens, revealed presence of FA in one case. FA islands were characterized by a fairly classic stellate reticulum surrounded by a single layer of tall, columnar, ameloblastic-like cells with nuclei at the opposite pole to the basement membrane. FAs were completely embedded in the connective tissue and they were not contiguous with the lumen of the lesions (Figs. 2,3).

**Figure 4:**
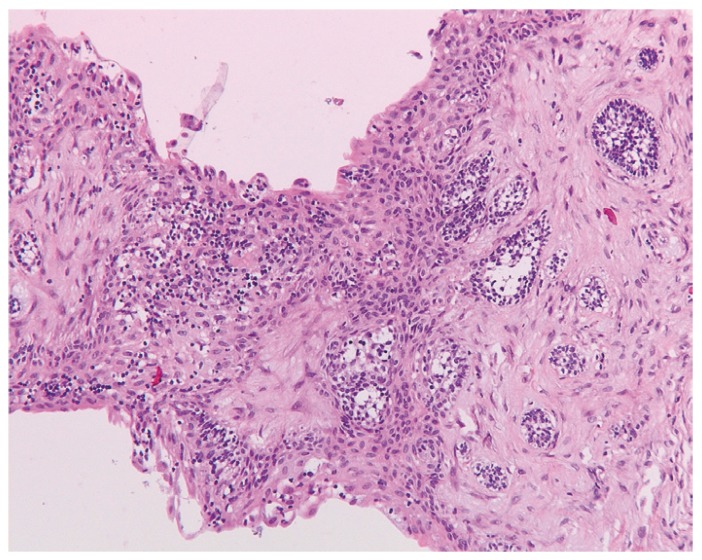
Ameloblastomatous changes in the wall of a dentigerous cyst (H.E.; orig.magn. x100).

In the group of DCs, the most frequently reported findings include the presence of a connective fibrous capsule lined by stratified squamous epithelium. Presence of chronic inflammatory infiltrate was reported in 7 cases while 3 cases presented myxoid changes in the connective tissue. One case was characterized by the presence of hyperplastic epithelium, while in another case, focal epithelial features of a keratocyst were reported. Actinomyces colonies were observed in one specimen. FAs were observed in 2 out of 7 cases re-evaluated because of the presence of OE in the connective tissue (Fig. 4). OE remnants were also reported in 5 “questionable” lesions.

Altogether, presence of small OE rests in the connective tissue was documented in 39 out of 164 (23.7%) specimens.

In no one of the 3 patients with FA, a (recurrent) ameloblastoma was registered in the PALGA database during a follow-up of 6 (case of FA in DF group), 8 and 22 years (cases of FAs in DC group).

## Discussion

Odontogenic tissue surrounding impacted teeth has the potential to differentiate in a wide variety of tissue types, including cystic and neoplastic tissue (5,13,14). The dentigerous cyst is the most frequent odontogenic lesion associated with unerupted teeth, followed by keratocyst, odontomas and ameloblastoma (14).

Radiological and histopathological criteria for distinguishing between a normal or slightly enlarged DF and a DC are controversial (1,6). Radiographical signs of DC include a pericoronal width of at least 3-4 mm and an asymmetric appearance of the radiolucency (1,6). Histopathological diagnosis of DC is mainly based on the presence of a continuous lining of stratified squamous epithelium. However, Adelsperger et al. showed that pericoronal tissue of 34% of 100 impacted third molars with a follicular space < 2mm were characterized by squamous epithelium indistinguable from the histologic changes found in DCs (15). Similarly, in a radiographical and histopathological evaluation of 1662 DCs and 824 DFs, Daley et al. pointed out that the separation of these entities is difficult and they concluded that a reliable distinction may only be based on the identification of a cystic cavity at the surgical operation (16). In the present evaluation, 15.4% of specimens showing histopathological features of DF, were submitted with a working diagnosis of DC and 62.5% of cases clinically diagnosed as DFs, were histopathologically classified as DCs.

The presence of OE islands in the connective pericoronal tissue of unerupted teeth is a well-known phenomenon (1,10). In a study on 847 DFs and dental papillas, Kim et al. found small OE remnants in the connective tissue in 79% of the specimens. No features of ameloblastic differentiation were observed in this series (1). In a similar analysis, Gorlin observed OE islands in 3% of 200 cases of DCs (14). According to Paul et al. about 82% of all follicular cysts contain OE in the cyst wall (11). In the present analysis, OE remnants in connective tissue were documented in 39 cases (23.7%). These structures are most numerous in younger persons and gradually decrease with age. Interestingly, Daley et al. documented that squamous differentiation of the OE islands may occur between the ages of 16 and 26 years (16).

It is generally thought that OE remnants are inactive and do not have clinical significance (10). However, it has been hypotesize that some ameloblastoma may develop within follicular structures with a possible origin from either the epithelial lining or the OE islands in connective tissue (5,10). In his series, Regezi reported an incidence of 0.14% of ameloblastoma development around impacted third molars and Stathopoulos in another study concluded that 0.27% of cases out of 417 specimens of tissue surrounding impacted wisdom teeth were associated to the same phenomenon (17,18).

According to the “World Health Organization (WHO) classification of head and neck tumours”, ameloblastomas with follicular pattern are histopathologically characterized by islands of odontogenic epithelium within a fibrous stroma. The basal cells of these islands are columnar, hyperchromatic and lined up in a palisaded fashion. The central cells may be loosely arranged, resembling stellate reticulum (19). However, in the WHO description it is not mentioned how many neoplastic islands are actually needed for classifying a lesion as ameloblastoma. The histopathological term “focal ameloblastoma” is here used to designate one or few epithelial islands frankly displaying ameloblastomatous features. Terms such as “mural ameloblastoma” and “proliferating odontogenic epithelium” have been used for describing a phenomenon apparently similar to those reported here (10).

The striking morphological and architectural similarities between OE islands of FA and ameloblastoma, would suggest that both the entities represent the same pathological process at different developmental stages. Particularly, FA might be regarded as an “early” ameloblastoma. However, on the basis of the data of the present evaluation, it is not possible to draw any conclusion on the nature of FA. The question whether these structures are separated nosological entities lacking neoplastic activity remains therefore unanswered.

FAs described in this report were completely embedded in the connective tissue, being localized away from the specimen margins. The surgical removal of these structures appears therefore radical. No (recurrent) ameloblastomas were registered in the PALGA database during a follow-up of 6 (FA in DF group), 8 and 22 years (FAs in DC group).

On the basis of our analysis it would seem reasonable to advocate the complete removal of pericoronal soft tissues during the extraction of impacted teeth.

Further analyses are required in order to clarify the intriguing and fascinating nature of FA.
